# Supplementary material for: Next-generation genetic sexing strain establishment in the agricultural pest Ceratitis capitata
Source: Sci Rep. 2023 Nov 14;13:19866. doi: 10.1038/s41598-023-47276-5 (PMC10646097; doi:10.1038/s41598-023-47276-5)
Supplement: Supplementary file 1 — Supplementary Legends. [file 41598_2023_47276_MOESM1_ESM.docx]

**Supplementary Figure 1: Inverse PCR integrations**

The map of integrations of the 4 unique strains for the 795H1 and 795K1 constructs. H-001 and H-002 strains harbor the 795H1 cassette, while K-001 and K-002 strains harbor the 795K1 cassette. The figure was constructed in RStudio.

**Supplementary Figure 2: Images of 795H1 and 795K1 homozygous females**

The homozygous females harboring the *Anastrepha ludens* *transformer* (*tra*) intron-containing 795K1 cassette have a weaker DsRed signal (left) compared to homozygous females harboring the *Ceratitis capitata* tra intron containing 795H1 cassette (middle). These were imaged alongside a wild-type female (right).

**Supplementary Figure 3: Images of transgenic and wild-type eggs and day 1 transgenic larvae**

The egg images showcase the wild-type eggs, the homozygous SEPARATOR eggs, and the homozygous SEPARATOR Day 1 larvae. The SEPARATOR eggs all express GFP and a variable degree of DsRed, whilst the day 1 male and female larvae can be differentiated by the complete absence of a DsRed signal in males.

**Supplementary Figure 4: DsRed is spliced sex-specifically in all transgenic strains**

Diagrams showcasing the expected sex-specific DsRed splicing patterns in (A) the 795H1-harboring and (B) 795K1-harboring flies via the *transformer* (*tra*) intron from (A) *Ceratitis capitata* (29) and (B) *Anastrepha ludens* (32) accordingly. Forward and reverse primers (Supplementary Table 5), specific to the exogenous elements of both constructs, used in the PCR amplification are shown in (A) and (B) as F and R, respectively. (C) and (D) are annotated electrophoresis gel images with amplification from male and female genomic DNA (gDNA), and male, and female cDNA. A DNA ladder was run in the left-most lanes and negative controls in the right-most lanes.

Supplementary Figure 5: The original gel showcasing DsRed is spliced sex-specifically in all transgenic strains

The original electrophoresis gel from Supplementary Figure 4 (C) and (D) of PCR products from male and female genomic DNA (gDNA), and male, and female cDNA. A DNA ladder was run in the left-most lanes and negative controls in the right-most lanes.

**Supplementary Table 1: G0 and G1 line raw data**

Injection summary for 795H1 and 795K1 constructs. G0 data includes the number of injected eggs, hatched larvae and surviving adults. G1 data showcases the numbers of marker-positive adults screened for GFP and DsRed fluorescence markers.

**Supplementary Table 2: Genomic integrations determined through inverse PCR**

Inverse PCR outcomes, visualized in Supplementary Figure 2, obtained through mapping sequencing outputs to GenBank GCA_905071925.1 *C. capitata* genome re-assembly summarized by construct.

**Supplementary Table 3: G9 and G10 line raw data**

All adult flies in the G9 and G10 generations were phenotyped by sex and screened for GFP and DsRed fluorescence markers.

**Supplementary Table 4: Egg laying and egg hatching raw data**

The unhatched eggs were counted via ImageJ twice, the first, immediately after collection, and then once more after 4 days. Number of hatched eggs was determined by calculating the difference between numbers of unhatched eggs in pictures 1 and 2.

**Supplementary Table 5: Primer summary**

The primers used for cloning of 795H1 and 795K1 constructs, and the primers used for DsRed splicing confirmation (Supplementary Figure 3).
