# Supplementary material for: Next-generation genetic sexing strain establishment in the agricultural pest Ceratitis capitata
Source: Sci Rep. 2023 Nov 14;13:19866. doi: 10.1038/s41598-023-47276-5 (PMC10646097; doi:10.1038/s41598-023-47276-5)
Supplement: Supplementary file 2 — Supplementary Information. [file 41598_2023_47276_MOESM2_ESM.pdf]

Next-generation genetic sexing strain establishment in the agricultural pest *Ceratitis capitata*

Serafima Davydova[1†], Junru Liu[2†], Nikolay P. Kandul[2], W. Evan Braswell[3], Omar S. Akbari[2], Angela Meccariello[1*]

1.Department of Life Sciences, Imperial College London, London SW7 2AZ, United Kingdom

2.School of Biological Sciences, Department of Cell and Developmental Biology, University of California, San Diego, La Jolla, CA 92093, United States of America

3.USDA APHIS PPQ Science and Technology Insect Management and Molecular Diagnostic Laboratory, 22675 North Moorefield Road, Edinburg, Texas 78541, United States of America

* Corresponding author

† S.D and J.L contributed equally to this work

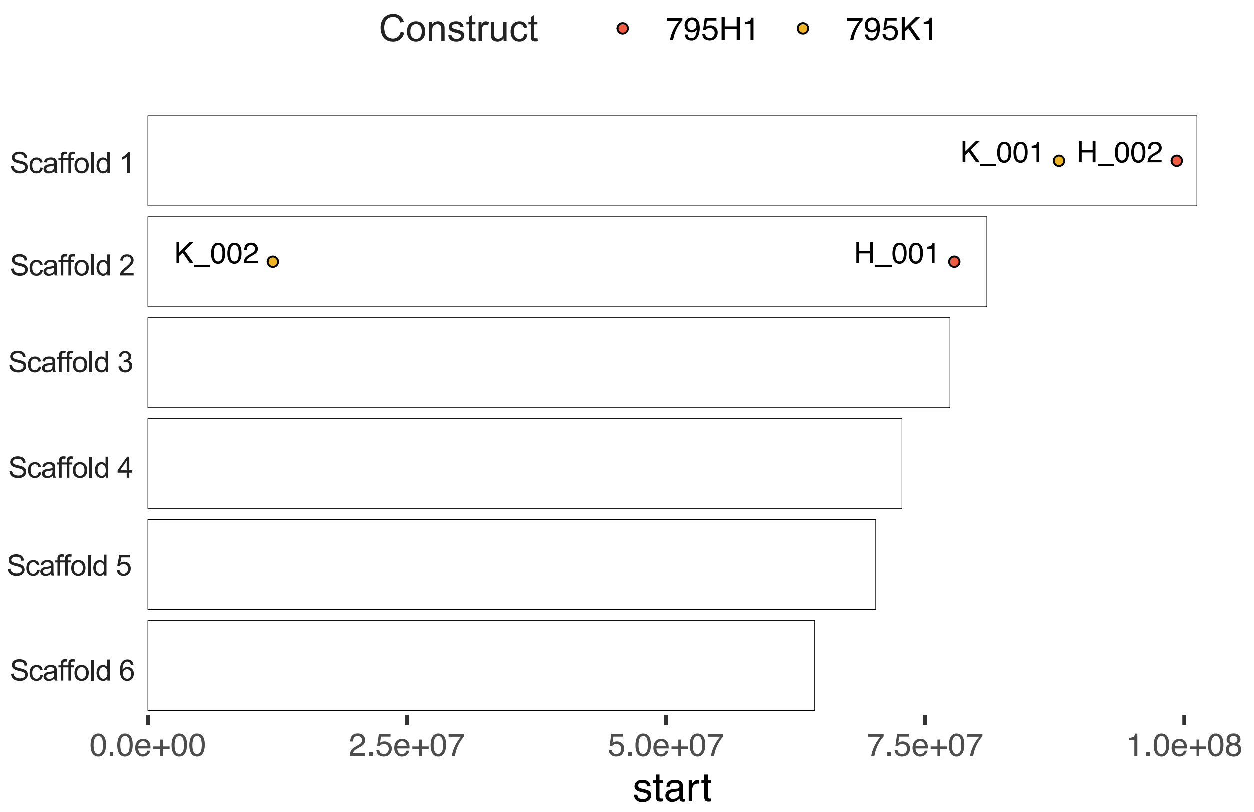

Supplementary Figure 1: Inverse PCR integrations

The map of integrations of the 4 unique strains for the 795H1 and 795K1 constructs. H-001 and H-002 strains harbor the 795H1 cassette, while K-001 and K-002 strains harbor the 795K1 cassette. The figure was constructed in RStudio.

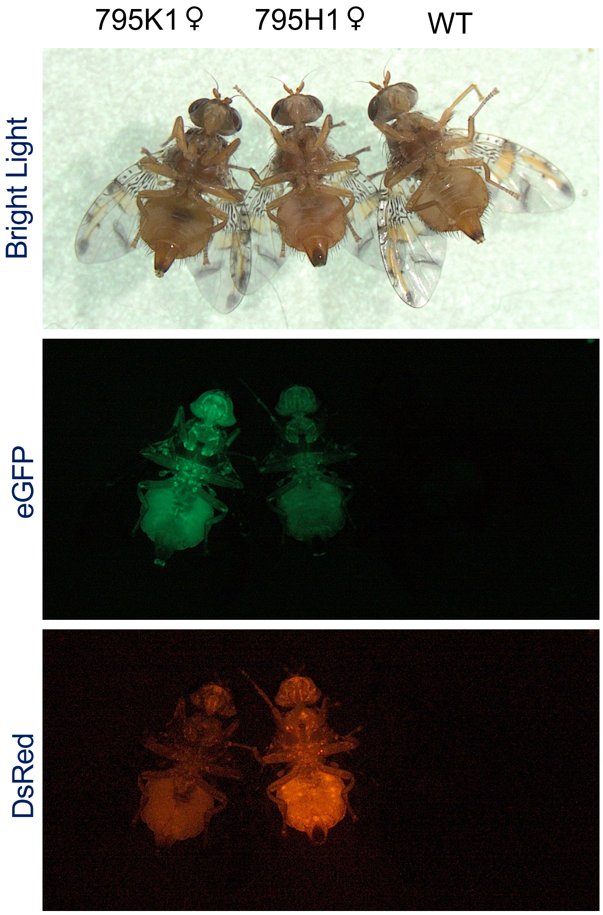

Supplementary Figure 2: Images of 795H1 and 795K1 homozygous females

The homozygous females harboring the *Anastrepha ludens transformer* (*tra*) intron-containing 795K1 cassette have a weaker DsRed signal (left) compared to homozygous females harboring the *Ceratitis capitata* tra intron containing 795H1 cassette (middle). These were imaged alongside a wild-type female (right).

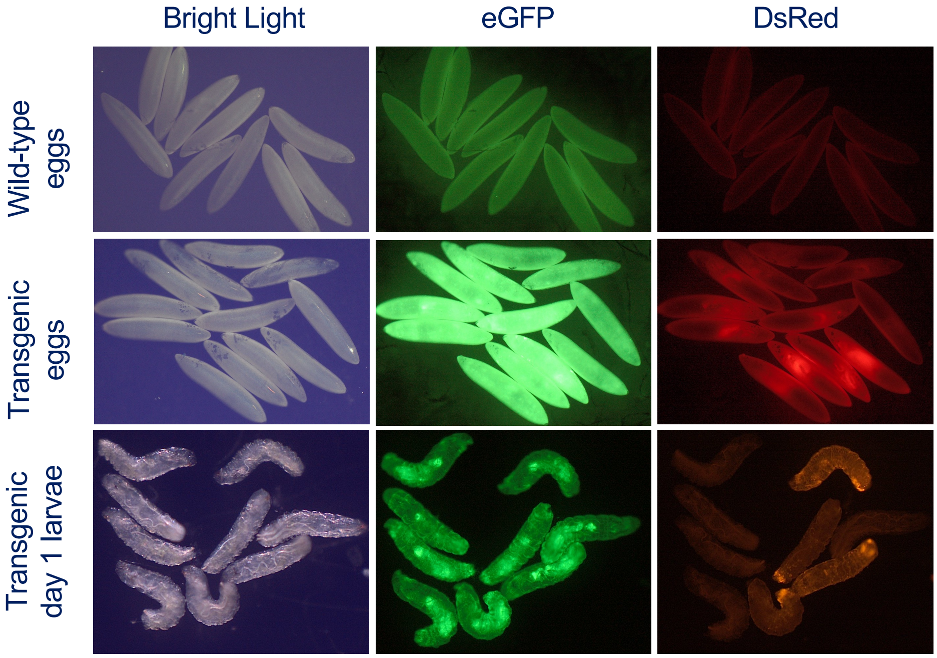

Supplementary Figure 3: Images of transgenic and wild-type eggs and day 1 transgenic larvae

The egg images showcase the wild-type eggs, the homozygous SEPARATOR eggs, and the homozygous SEPARATOR day 1 larvae. The SEPARATOR eggs all express GFP and a variable degree of DsRed, whilst the day 1 male and female larvae can be differentiated by the complete absence of a DsRed signal in males.

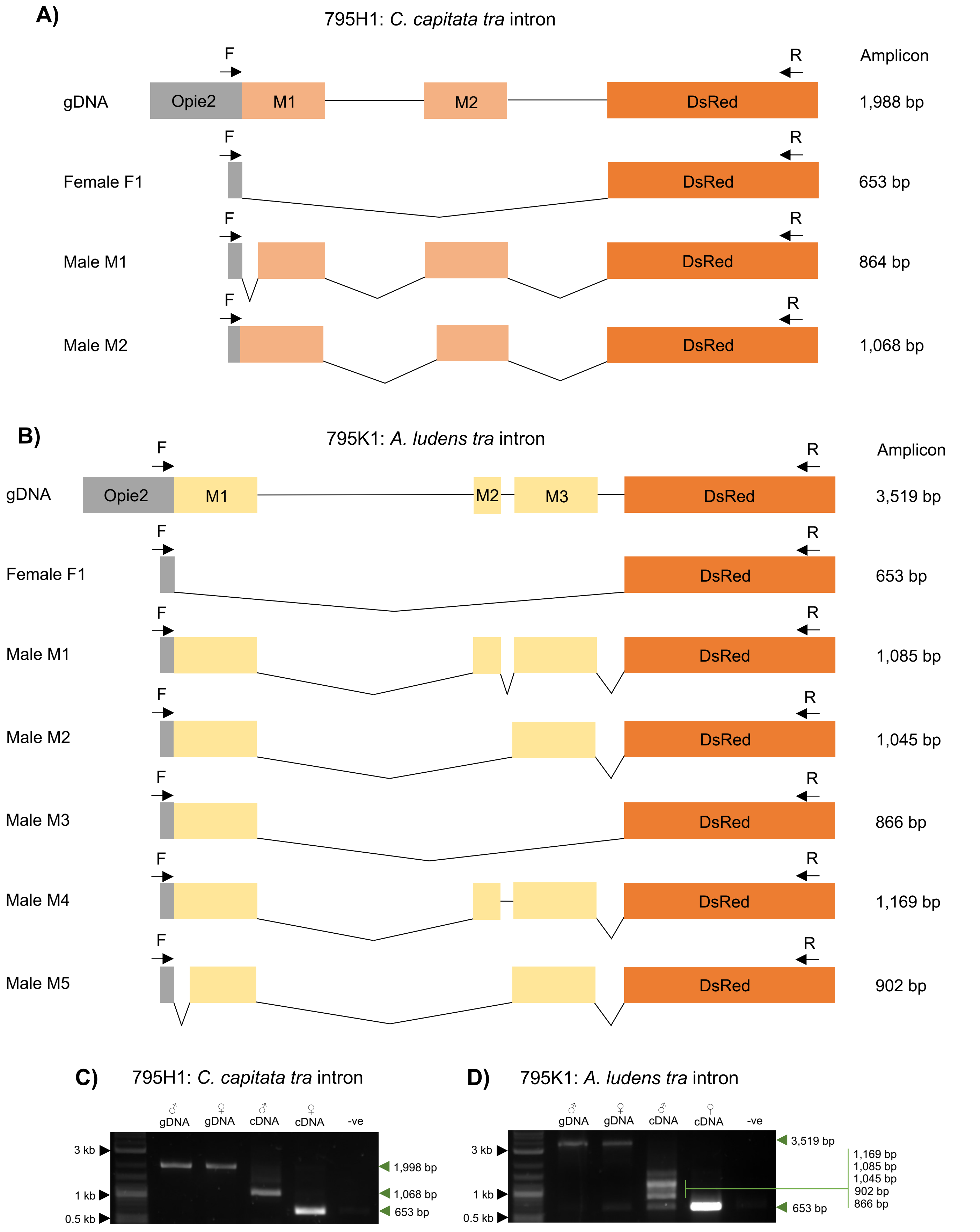

Supplementary Figure 4: DsRed is spliced sex-specifically in all transgenic strains

Diagrams showcasing the expected sex-specific DsRed splicing patterns in (A) the 795H1-harboring and (B) 795K1-harboring flies via the *transformer* (*tra*) intron from (A) *Ceratitis capitata* (29) and (B) *Anastrepha ludens* (32) accordingly. Forward and reverse primers (Supplementary Table 5), specific to the exogenous elements of both constructs, used in the PCR amplification are shown in (A) and (B) as F and R, respectively. (C) and (D) are annotated electrophoresis gel images with amplification from male and female genomic DNA (gDNA), and male, and female cDNA. A DNA ladder was run in the left-most lanes and negative controls in the right-most lanes.

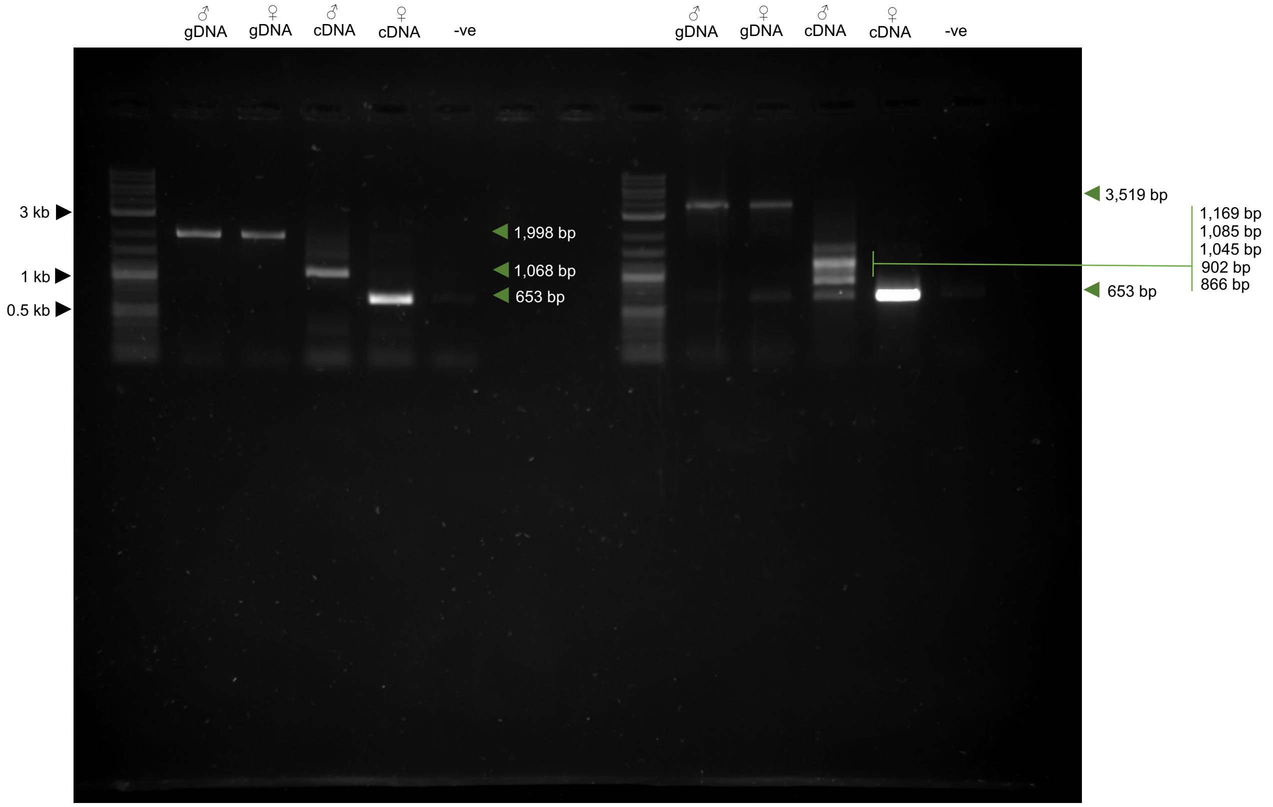

Supplementary Figure 5: The original gel showcasing DsRed is spliced sex-specifically in all transgenic strains
The original electrophoresis gel from Supplementary Figure 4 (C) and (D) of PCR products from male and female genomic DNA (gDNA), and male, and female cDNA. A DNA ladder was run in the left-most lanes and negative controls in the right-most lanes.

Supplementary Table 1: G0 and G1 line raw data

Injection summary for 795H1 and 795K1 constructs. G0 data includes the number of injected eggs, hatched larvae and surviving adults. G1 data showcases the numbers of marker-positive adults screened for GFP and DsRed fluorescence markers.

Transformation Construct	Eggs Injected	G0 Hatched Larvae	G0 Adults	G1 DsRed+ /GFP+ ♀	G1 DsRed- /GFP+ ♂
795H1	250	210	84	31	11
795K1	250	141	52	4	2

Supplementary Table 2: Genomic integrations determined through inverse PCR

Inverse PCR outcomes, visualized in Supplementary Figure 2, obtained through mapping sequencing outputs to GenBank GCA_905071925.1 *C. capitata* genome re-assembly summarized by construct.

Transformation Construct	Subline	Scaffold	Annotation	Integration Site
**795H1**	H-001	2	77 806 718	CACATCTCTA**TTAA-PB-TTAA**CAGCTATAAT
	H-002	1	99 273 433	GTAAAACTTT**TTAA-PB-TTAA**AACCAATTCA
**795K1**	K-001	1	87 907 601	CCAGCAGTGA**TTAA-PB-TTAA**CGTAGGTACG
	K-002	2	12 057 725	TTGGCAACTG**TTAA-PB-TTAA**ACAGACATCA

Supplementary Table 3: G9 and G10 line raw data

All adult flies in the G9 and G10 generations were phenotyped by sex and screened for GFP and DsRed fluorescence markers.

Transformation Construct	Subline	DsRed+/GFP+ ♀	DsRed-/GFP+ ♂
**795H1**	H-001	167	221
		249	231
	H-002	156	219
		216	229
**795K1**	K-001	106	105
		243	227
	K-002	330	307
		380	401

Supplementary Table 4: Egg laying and egg hatching raw data

The unhatched eggs were counted via ImageJ twice, the first, immediately after collection, and then once more after 4 days. Number of hatched eggs was determined by calculating the difference between numbers of unhatched eggs in pictures 1 and 2.

Construct	Strain	Replicate	Embryos	Hatched larvae
N/A	WT	1	171	133
		2	164	134
		3	210	173
795H1	H-001	1	249	212
		2	88	73
		3	211	161
	H-002	1	50	42
		2	132	98
		3	110	93
795K1	K-001	1	104	44
		2	89	52
		3	225	90
	K-002	1	146	81
		2	253	179
		3	246	161

Supplementary Table 5: Primer summary

The primers used for cloning of 795H1 and 795K1 constructs, and the primers used for DsRed splicing confirmation (Supplementary Figure 3).

Purpose	Name	Sequence (5'-3')
C. capitata intron	825A	AAAAATAAGGTCAGCAGCCATGAC
	825B	GTCATGGCTGCTGACCTTATTTTT
	825C	AGACATTCCAAATTCAAGTTAACAAATTAAT
	825D	ATTAATTTGTTAACTTGAATTTGGAATGTCT
A. ludens intron	1168-Tra-gDNA-R1	CTTGCTCTGGAGTCCGTTAAA
	1168-Tra-gDNA-F2	CCTGAGCAGATAACCCTCTTTC
	1168-Tra-gDNA-F3	GGTCGTTTCGGTAACGTAGAA
	1168-Tra-gDNA-R2	GTCATCAGTTGTCGGCATTTAC
DsRed splicing PCR	Opie2_V2	CCGCAACCTGTCTCTGGTG
	DsRed_B	CGATGGTGTAGTCCTCGTTGTG
